# Loss of tapasin correlates with diminished CD8^+^ T-cell immunity and prognosis in colorectal cancer

**DOI:** 10.1186/s12967-015-0647-1

**Published:** 2015-08-27

**Authors:** Lena Sokol, Viktor H. Koelzer, Tilman T. Rau, Eva Karamitopoulou, Inti Zlobec, Alessandro Lugli

**Affiliations:** Translational Research Unit (TRU), Institute of Pathology, University of Bern, Murtenstrasse 31, 3010 Bern, Switzerland; Clinical Pathology Division, Institute of Pathology, University of Bern, Murtenstrasse 31, 3010 Bern, Switzerland

**Keywords:** Colorectal cancer, Prognostic factor, Immune infiltration, Tapasin, MHC I, Antigen presentation, CD8, Cytotoxic lymphocytes, Immune activation, Immunosurveillance

## Abstract

**Background:**

Tapasin is a crucial component of the major histocompatibility (MHC) class I antigen presentation pathway. Defects in this pathway can lead to tumor immune evasion. The aim of this study was to test whether tapasin expression correlates with CD8^+^ cytotoxic T lymphocyte (CTL) infiltration of colorectal cancer (CRC) and overall survival.

**Methods:**

A next-generation tissue microarray (ngTMA) of 198 CRC patients with full clinicopathological information was included in this study. TMA slides were immunostained for tapasin, MHC I and CD8. Marker expression was analyzed with immune-cell infiltration, patient survival and TNM-staging.

**Results:**

A reduction of tapasin expression strongly correlated with venous invasion (AUC 0.682, OR 2.7, p = 0.002; 95 % CI 1.7–5.0), lymphatic invasion (AUC 0.620, OR 2.0, p = 0.005; 95 % CI 1.3–3.3), distant metastasis (AUC 0.727, OR 2.9, p = 0.004; 95 % CI 1.4–5.9) and an infiltrative tumor border configuration (AUC 0.621, OR 2.2, p = 0.017; 95 % CI 1.2–4.4). Further, tapasin expression was associated with CD8^+^ CTL infiltration (AUC 0.729, OR 5.4, p < 0.001; 95 % CI 2.6–11), and favorable overall survival (p = 0.004, HR 0.6, 95 % CI 0.42–0.85).

**Conclusions:**

Consistent with published functional data showing that tapasin promotes antigen presentation, as well as tumor immune recognition and destruction by CD8^+^ CTLs, a reduction in tapasin expression is associated with tumor progression in CRC.

**Electronic supplementary material:**

The online version of this article (doi:10.1186/s12967-015-0647-1) contains supplementary material, which is available to authorized users.

## Background

The ability of the immune system to recognize and attack tumor cells is being acknowledged as an increasingly important factor in overall disease progression [1–3]. Specifically, in colorectal cancer (CRC), increased tumor infiltration by CD4^+^ and CD8^+^ T-lymphocytes (CTLs) correlates positively with overall (OS) and disease-free survival (DFS) [2].The surface presentation of antigenic peptides by the Major Histocompatibility Complex class I molecules (MHC I) is indispensable for the initiation of the CD8^+^ T-lymphocyte anti-tumoral immune response. The MHC I consists of the heavy α-chain (HLA) and a β_2_-microglobulin (B2M) chain. Folding and assembly is assisted by the chaperone calnexin. The antigenic peptides are generated by the proteasome and loaded into the assembled MHC I molecule through a multi-step process involving (1) translocation of the peptides into the endoplasmic reticulum by TAP1/2 proteins, and (2) binding of the peptides to assembled MHC I molecules facilitated by the peptide loading complex (TAP1, TAP2, calreticulin, ERp57 and tapasin). A conformational change in the molecule resulting from peptide binding releases the MHC I from the loading complex and allows surface presentation of the antigen [4, 5].

Tapasin is an essential member of the MHC I pathway. On a molecular level, it is a transmembrane glycoprotein which forms a stable heterodimer with the thiol oxidoreductase ERp57 [6]. Cells missing tapasin have reduced MHC I surface expression [7], and display MHC I molecules loaded with suboptimal, low affinity peptides [8], resulting in decreased CTL recognition [9]. Loss of tapasin therefore contributes to reduced immunogenicity and immune evasion of tumors [10]. The importance of tapasin for antigen presentation in cancer was first shown in vivo in mouse tumor models. Mice were injected with a lung carcinoma cell line in which many components of the MHC I pathway were downregulated. Transfection of tapasin on this background was sufficient to restore antigen presentation, increase the antigen-specific immune response, reduce tumor growth, and increase survival [11]. However, the potential effect of tapasin on patient outcome in human CRC has not been determined to date. The aim of this study was therefore to test whether tapasin expression correlates with the degree of CTL infiltration and overall survival in CRC and thus influences overall survival.

## Methods

### Patients

This study was designed to comply with the reporting recommendations for tumor marker prognostic studies (REMARK) guidelines for tumor marker prognostic studies. The study design is shown in Additional file 1: Figure S1. 220 non-consecutive surgically treated CRC patients treated from 2004 to 2007 at the Areteiaion University Hospital, University of Athens, Greece, were retrospectively included in this study. Clinical data were obtained from patient records including patient age at diagnosis, gender, tumor location and diameter, and overall survival time. An experienced GI pathologist (EK) reviewed all histomorphological data of the surgical resections, and recorded data on pTNM classification, tumor grade, lymphatic and venous invasion, histological subtype and tumor border configuration. Clinicopathological features of the 198 patients for which tapasin expression could be analyzed are listed in Additional file 2: Table S1. The median survival time was 58 months (95 % CI 50.9–65.1 month). Detailed clinicopathological data for this cohort has been published [12]. The use of patient data has been approved by the local Ethics Committee of the University of Athens, Greece.

### Assay methods

Using a digital pathology and automated tissue microarraying approach, a next-generation tissue microarray (ngTMA [13]) was constructed that included tissue spots from the tumor center (n = 2), tumor front (n = 2) and matched normal colorectal mucosa (n = 1) from 220 patients [13]. The ngTMA blocks were sectioned at 4 µm and stained for MHC I with an established antibody [14] that detects the main human HLA types A, B and C (Abcam #ab70328, dilution 1:4000, pre-treatment citrate 30′, 100 °C), for tapasin (Novus #NBP1-86968, dilution 1:50, pre-treatment tris 30′, 95 °C) and CD8 (Dako, #M7103, dilution 1:100, pre-treatment tris 20′, 90 °C) by automated immunohistochemistry using a standard protocol on a LEICA Bond-III.

### Evaluation of immunohistochemistry

To detect the MHC I complex, we evaluated the percentage of cells which showed membranous expression of the classical heavy α-chain isoforms (HLA-ABC) in relation to the total number of cells in the spot under high power (400×) magnification. The expression varied between 0 and 100 percent, with the median and mean scores being 30 and 23.8, respectively. Intratumoral lymphocytes and normal mucosa served as an internal positive control. CD8^+^ CTLs were counted in each spot. Because tapasin is ubiquitously expressed, immunoreactivity was assessed by scoring the staining intensity. The intensity was scored as 0–3, with 0 representing complete absence of marker reactivity, 1 as low staining intensity visible at 200×, 2 as medium staining intensity visible at 100×, 3 as high staining intensity visible at all magnifications, based on an adaptation of the well-established protocol for the assessment of HER2 biomarker expression by Rüschoff and colleagues [15]. The expression varied between 0 and 3 with the median and mean scores being 2.31 and 2.33, respectively. Normal mucosa served as an internal positive control.

### Statistics

The association of protein markers with clinicopathological features was analyzed by receiver operating characteristic (ROC) curve analysis and logistic regression. The p values, odds ratios (OR) and 95 % CI for each analysis were obtained, as well as the area under the curve (AUC), with values closer to 1 indicating a better discriminatory ability for binary end point. For survival assessment using non-dichotomized data, Cox regression analyses were performed. After verification of the proportional hazards assumption, multivariable Cox regression analysis was carried out using pT (primary tumor), pN (regional lymph nodes), pM (distant metastasis), adjuvant therapy (postoperative therapy, such as chemotherapy, radiotherapy or combination therapies), CD8^+^ CTL infiltration and protein expression as possible confounding factors. Hazard ratios (HR) and 95 % CI were used to determine the effect size. Differences in survival time were displayed using standard Kaplan–Meier curves and tested using the log-rank test in univariate analysis. The time of survival was defined as the time of an event occurrence (death) or censored (patient lost to follow-up) relative to the date of operation. Analyses were performed using SPSS Version 21.

## Results

### Analysis of tapasin and MHC I expression patterns in normal mucosa and CRC by immunohistochemistry

**Fig. 1:**
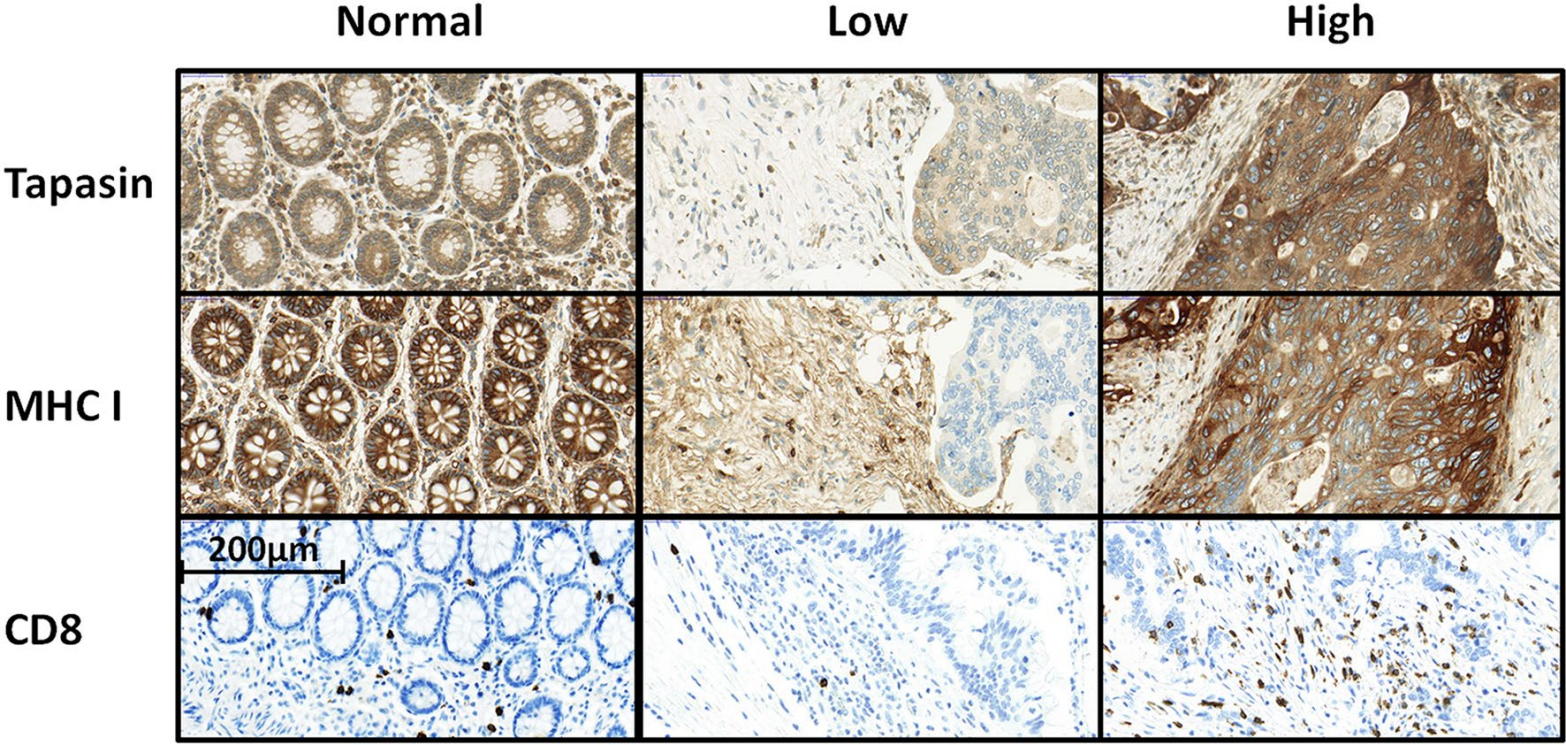
Immunohistochemistry staining. Analysis of HLA-ABC (MHC I), Tapasin, and CD8 staining in normal tissue and high and low intensity staining in tumor tissue

Tapasin expression in normal tissue was low to moderate; the protein showed a cytoplasmic/membranous expression. The staining was diffuse and homogeneous in the majority of normal and tumor spots. MHC I was downregulated (score lower than the overall mean) in 50.7 % of cases in normal mucosa and in 60 % cases of CRC tumors. Tapasin could be detected in the normal mucosa of 93 % of cases, and was downregulated in 48 % of tumors (p = 0.002). Representative IHC stainings can be seen in Fig. 1. Interestingly, only 2/19 available metastatic cases showed tapasin expression (p = 0.002).

### Association of tapasin expression with clinicopathological features and survival in CRC

Reduced tapasin expression was associated with venous invasion (AUC 0.682, p = 0.002, OR 2.70; 95 % CI 1.72–5.0), lymphatic invasion (AUC 0.620, p = 0.005, OR 2.04; 95 % CI 1.25–3.33), and the presence of distant metastasis (AUC 0.727, p = 0.004, OR 2.86; 95 % CI 1.41–5.88). Low tapasin was also concurrent with an infiltrative tumor border configuration (AUC 0.621 p = 0.017, OR 2.22; 95 % CI 1.15–4.35). The associations of tapasin with these and other clinicopathological features are listed in Table 1.

**Table 1:** Univariate analysis: association of tapasin decrease with clinicopathological features

	P-value	OR	95 % CI	AUC
Lymphatic invasion	0.005	2.04	1.25–3.33	0.62
Venous invasion	0.002	2.70	1.72–5.00	0.68
pT	0.882	1.04	0.61–1.79	0.50
pN	0.060	1.59	0.98–2.56	0.58
pM	0.004	2.86	1.41–5.88	0.73
Intratumoral CD8^+^ CTL	<0.001	0.19	0.09–0.38	0.73
Tumor border configuration	0.017	2.22	1.15–4.35	0.62
Tumor grade	0.280	1.30	0.81–2.13	0.61
Gender	0.466	0.84	0.53–1.33	0.55
Adjuvant therapy	0.578	1.15	0.70–1.85	0.49

### Univariate and multivariate survival analysis

**Fig. 2:**
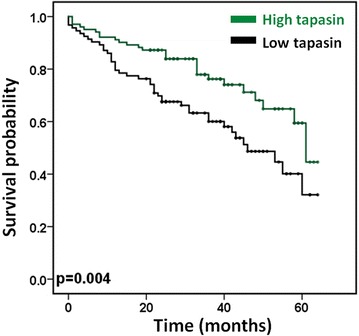
Kaplan–Meier survival analysis of differential tapasin expression. Patients with high tapasin levels detected in the primary tumor have a higher 5-year survival rate (*Black* low tapasin: 0.321 ± 0.094, *green* high tapasin: 0.446 ± 0.142)

High tapasin expression in the tumor was found to be a significant favorable prognostic factor (p = 0.004, HR 0.6, 95 % CI 0.42–0.85, Table 2). To visualize this effect, we dichotomized the tapasin values using the mean expression score to differentiate between 95 tapasin negative and 103 tapasin positive cases and plotted a Kaplan–Meier curve (Fig. 2). The favorable prognostic effect of tapasin was independently maintained in multivariate analysis when adjusting for potential confounder factors such as patient age and gender, tumor grade, pT, tumor size and location, and adjuvant therapy (p = 0.021). Nevertheless, this effect was lost when pN and pM were added into the analysis (p = 0.327, Table 3).

**Table 2:** Cox regression analysis of survival—univariate analysis of single factors

		HR	95 % CI	P-value
Tapasin	Baseline	1.00		
	By intensity	0.60	0.42–0.85	0.004
pT	pT1-2	1.00		
	pT3-4	2.74	1.31–5.73	0.007
pN	pN0	1.00		
	pN1-2	4.14	2.39–7.18	<0.001
pM	pM0	1.00		
	pM1	6.08	3.38–10.92	<0.001
Adjuvant therapy	None	1.00		
	Tx	1.48	0.89–2.48	0.143
Intratumoral CD8^+^ CTL	Low	1.00		
	High	0.28	0.13–0.60	0.001

**Table 3:** Multivariable Cox regression analysis for tapasin with TNM stage as confounding factors

Factors	HR	95 % CI	P-value
Tapasin			
Baseline	1		
By intensity	0.83	0.56–1.21	0.327
pT			
pT1-2	1		
pT3-4	1.38	0.62–3.05	0.431
pN			
pN0	1		
pN1-2	3.04	1.79–6.64	<0.001
pM			
pM0	1		
pM1	3.512	1.88–6.54	<0.001

### Tapasin predicts CD8^+^ CTL tumor infiltration

Next we tested the ability of tapasin expression to predict intratumoral CD8^+^ CTL invasion, as well as the odds of CTL tumor invasion in the presence and absence of tapasin. Interestingly, a significantly higher presence of intratumoral CD8^+^ CTLs was found in tapasin-high tumors (AUC 0.729, p < 0.001, OR 5.4; 95 % CI 2.6–11 and AUC 0.650, p = 0.002, OR 2.4 95 % CI 1.4–4.2, respectively, Table 1). Tapasin also increases the likelihood of detecting membranous MHC I expression in tumors by up to two-fold (p = 0.035, OR 1.729, 95 % CI 1.04–2.88). Interestingly, the effects of tapasin on CD8^+^ CTL tumor infiltration were independent of MHC I membrane expression (p = 0.008, OR 0.615, 95 % CI 0.429–0.882).

### Association of tapasin expression and CD8^+^ CTL infiltration with survival in CRC

To assess whether the prognostic effect of tapasin can be seen as independent of CD8^+^ infiltration, we added it as a confounder in the Cox regression analysis. Under these conditions, tapasin lost its prognostic effect (p = 0.117). Additionally, we could see no benefit of a combined marker approach (tapasin and CD8^+^ CTL infiltration, data not shown).

## Discussion

The aim of this study was to characterize the expression of tapasin as a potential prognostic tumor marker in CRC. We show that tapasin is decreased in invasive CRC, with this effect being even more pronounced in metastatic tumors. This is consistent with a previous study of tapasin expression in CRC and matched normal tissue, where gradual and increasing tapasin loss was likewise detected with tumor progression [16]. Expression of tapasin is also decreased in many other human cancers, including ovarian carcinoma, melanoma, glioblastoma, and salivary gland cancer [16–20]. We could furthermore correlate reduced tapasin expression with markers of increased invasiveness and systemic spread of the tumor, characterized by increased venous and lymphatic invasion, as well as distant metastasis. Importantly, we identify a strong survival advantage of patients bearing tapasin-positive tumors. Data from other groups have also shown similar consequences of tapasin decrease—in ovarian cancer it has been linked to higher stage, positive lymph nodes and considerably shorter survival time [17]. Likewise, in glioblastoma and salivary gland cancer, reduced tapasin expression correlated with shorter survival times [19, 20]. However, in our study, the prognostic effect of tapasin was lost in a multivariate analysis, indicating that tapasin does not contribute independent information to a prognosis. Lastly we show that tapasin expression correlated with increased membranous staining of MHC I, and as a possible consequence, we detected a drastic increase of intratumoral CD8^+^ CTLs in tapasin-positive tumors. Interestingly, the effect of tapasin on both CD8^+^ tumor infiltration and survival is independent of the amount of membranous MHC I. However, this result may be supported by multiple studies showing that tapasin expression not only promotes MHC I cell surface expression but that it increases total antigen presentation efficacy by ensuring the loading of a wide range of stable, highly affine peptides into the MHC I complex [21–24]. Consistent with these data, in a functional mouse study, Lou et al. demonstrated that tapasin expression restored susceptibility of tumor cells to CTL killing, and that animals with tapasin-expressing tumors had increased CD8^+^ CTL tumor infiltration and better survival [11]. As increased tumor infiltration by CD8^+^ CTLs has been demonstrated to strongly correlate with survival in CRC [2], we tested whether the prognostic effect of tapasin might be a reflection of its correlation with tumor immune invasion. Indeed, when CD8^+^ CTL infiltration was added as a confounding factor in a multivariate Cox regression analysis, tapasin did not retain its prognostic effect. These results suggests that the favorable survival effect of tapasin might be mediated both through an increase in antigen presentation quality and quantity. Tapasin expression thereby leads to the activation of the anti-tumoral immune response through increased recognition and infiltration of the tumor by CD8^+^ CTLs.

The current study has several strengths. It conforms to the criteria for reporting recommendations for tumor marker studies (REMARK guidelines [25]). The analyses have been performed using a very well characterized patient cohort for which full clinicopathological data is available, as well as information on treatment and overall survival. Protein expression was assessed using two tumor center and two tumor front punches in an ngTMA setup, ensuring equal staining conditions for all samples. The main weakness of this study is the lack of mechanistic data. However, the literature on tapasin in the context of immune recognition includes multiple comprehensive in vitro and in vivo functional studies which test the proposed molecular interactions. Therefore, this study focused on evaluating tapasin as a potential prognostic tumor marker in a translational setting.

## Conclusions

To conclude, consistent with published functional studies linking tapasin to efficient antigen presentation and tumor immune recognition by CD8^+^ CTLs, reduced expression of tapasin is associated with tumor progression in CRC. However, our understanding of the role of tapasin in CRC might benefit from testing its expression at the very interface of tumor and immune cells within the tumor microenvironment. MHC I-mediated antigen presentation is frequently downregulated during single cell invasion [14, 26, 27]. Potentially the quality of antigen presentation is also influenced by the conformation of HLA I molecules on the cell surface. Therefore, future studies could include evaluating the functional role and relevance of tapasin expression and MHC I conformation during single cell invasion and its correlation to the immune cell activation in the tumor microenvironment of CRC.

## Additional files

Additional file 1:
**Figure S1.** Study design, including patient number, and clinical and histopathological information overview. 220 CRC patients with full clinicopathological information were entered into the study. The association of tapasin with clinicopathological features and MHC I were analyzed using a multi-punch next generation tissue microarray. Additional file 2:
**Table S1.** Patient characteristics (colorectal cancer patient cohort, n = 198, max). Clinicopathological features of the 198 patients for which tapasin expression could be analyzed.

## Abbreviations

IHC: immunohistochemistry
CRC: colorectal cancer
CD8^+^ CTLs: CD8 positive cytotoxic T-lymphocytes
AUC: area under the curve
ROC: receiver operating characteristic
HLA: human leukocyte antigen
MHC: major histocompatibility complex
