# Supplementary figures and images for: Loss of tapasin correlates with diminished CD8+ T-cell immunity and prognosis in colorectal cancer
Source: J Transl Med. 2015 Aug 27;13:279. doi: 10.1186/s12967-015-0647-1 (PMC4551690; doi:10.1186/s12967-015-0647-1)

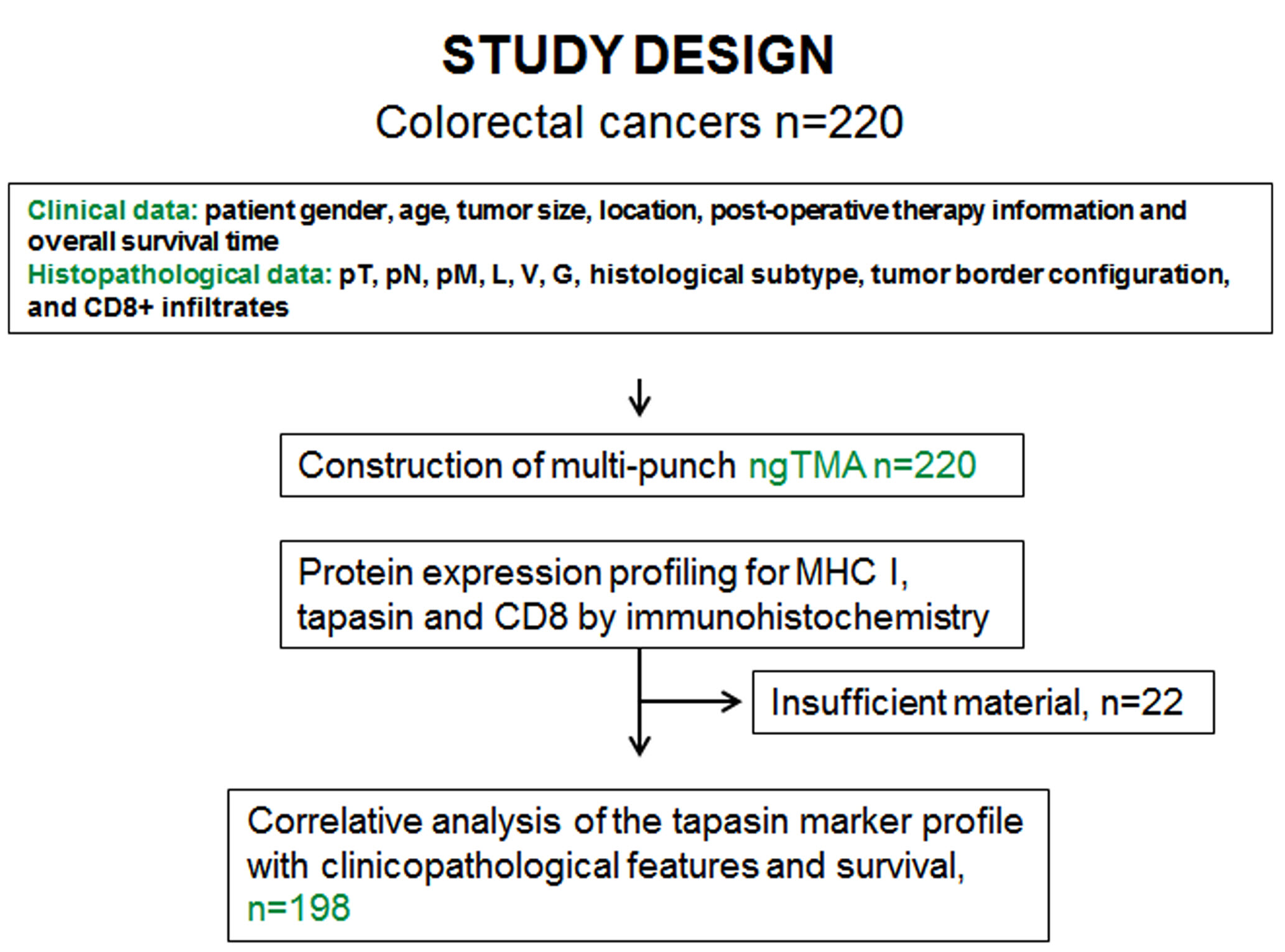

Supplement: Additional file 1: — Figure S1. Study design, including patient number, and clinical and histopathological information overview. 220 CRC patients with full clinicopathological information were entered into the study. The association of tapasin with clinicopathological features and MHC I were analyzed using a multi-punch next generation tissue microarray. [file 12967_2015_647_MOESM1_ESM.jpeg]
