# Supplementary material for: Loss of tapasin correlates with diminished CD8+ T-cell immunity and prognosis in colorectal cancer
Source: J Transl Med. 2015 Aug 27;13:279. doi: 10.1186/s12967-015-0647-1 (PMC4551690; doi:10.1186/s12967-015-0647-1)
Supplement: Additional file 2: — Table S1. Patient characteristics (colorectal cancer patient cohort, n = 198, max). Clinicopathological features of the 198 patients for which tapasin expression could be analyzed. [file 12967_2015_647_MOESM2_ESM.docx]

**Table S1: Patient characteristics (colorectal cancer patient cohort, n=198, max)**

| **Features** |  | **Frequency N (%)** |
| --- | --- | --- |
|  |  |  |
| **Age (yrs) (n=191)** | Median (min, max) | 68.85 (35.0, 93.0) |
|  |  |  |
| **Gender (n=195)** | Male | 93 (47.7) |
|  | Female | 102 (52.3) |
|  |  |  |
| **Histological subtype (n=195)** | Mucinous | 21 (10.8) |
|  | Non-mucinous | 174 (89.2) |
| **Tumor location (n=195)** | Left | 116 (59.5) |
|  | Rectum | 25 (12.8) |
|  | Right | 54 (27.7) |
|  |  |  |
| **pT classification (n=195)** | pT1 | 12 (6.2) |
|  | pT2 | 37 (19.0) |
|  | pT3 | 112 (57.4) |
|  | pT4 | 34 (17.4) |
|  |  |  |
| **pN classification (n=195)** | pN0 | 102 (52.3) |
|  | pN1-2 | 93 (47.7) |
|  |  |  |
| **Positive lymph nodes (n=194)** | Median (min, max) | 0 (0, 24) |
|  |  |  |
| **Metastasis (diagnosis) (n=195)** | M0 | 176 (90.3) |
|  | M1 | 19 (9.7) |
|  |  |  |
| **TNM stage (n=195)** | I | 41 (21.0) |
|  | II | 57 (29.2) |
|  | III | 78 (40.0) |
|  | IV | 19 (9.7) |
| **Tumor grade (n=195)** | G1-2 | 126 (64.6) |
|  | G3 | 69 (35.4) |
|  |  |  |
| **V classification (n=198)** | V0 | 165 (83.3) |
|  | V1-2 | 33 (16.7) |
|  |  |  |
| **L classification (n=196)** | L0 | 121 (61.7) |
|  | L1-2 | 75 (38.3) |
|  |  |  |
| **Post-operative therapy (n=197)** | None | 125 (63.5) |
|  | Yes | 72 (36.5) |
|  |  |  |
| **Survival (n=195)** | Alive/Censored | 125 (64.1) |
|  | Death | 70 (35.9) |
|  |  |  |
| **Overall survival time (n=195)** | 5-year (%) | 45.2 |
